# Effects of the vascular endothelial growth factor receptor-2 (VEGFR-2) inhibitor SU5416 on *in vitro* cultures of *Plasmodium falciparum*

**DOI:** 10.1186/1475-2875-13-201

**Published:** 2014-05-28

**Authors:** Casper Hempel, Nils Hoyer, Trine Staalsø, Jørgen A Kurtzhals

**Affiliations:** 1 Centre for Medical Parasitology, Department of Clinical Microbiology, Copenhagen University Hospital, Copenhagen, Denmark; 2 Department of International Health, Immunology and Microbiology, University of Copenhagen, Copenhagen, Denmark

**Keywords:** *Plasmodium falciparum*, Vascular endothelial growth factor, VEGF, Vascular endothelial growth factor receptor, Tyrosine kinase inhibitor, *Plasmodium berghei* ANKA

## Abstract

**Background:**

Vascular endothelial growth factor (VEGF) is taken up by parasitized red blood cells during malaria and stimulates intra-erythrocytic growth of *Plasmodium falciparum in vitro*. The cause and consequence of this uptake is not understood.

**Methods:**

*Plasmodium falciparum* was cultured *in vitro.* Parasite growth and intracellular VEGF levels were assessed using flow cytometry. Intracellular VEGF was visualized by fluorescence immunocytochemistry. Phosphorylated tyrosine was measured by western blotting. *In vivo* assessment of intra-erythrocytic VEGF was performed in *Plasmodium berghei* ANKA-infected C57BL/6 mice.

**Results:**

VEGF accumulated intracellularly in infected red blood cells, particularly in schizonts. *In vitro* growth of *P. falciparum* was unchanged when co-cultured with the anti-VEGF antibody bevacizumab or with an anti-VEGF receptor-1 peptide. In contrast, the VEGF receptor-2 inhibitor, SU5416, dose-dependently inhibited growth. None of the treatments reduced intracellular VEGF levels. Thus, the anti-parasitic effect of SU5416 seemed independent of VEGF uptake. SU5416 reduced phosphorylated tyrosine in parasitized red blood cells. Similarly, the broad-spectrum tyrosine kinase inhibitor genistein dose-dependently inhibited *P. falciparum* growth and reduced tyrosine phosphorylation. Neither bevacizumab nor anti-VEGF receptor-1 peptide affected tyrosine kinase activity. Finally, *in vivo* uptake of VEGF in *P. berghei* ANKA was demonstrated, analogous to the *in vitro* uptake in *P. falciparum,* making it a possible model for the effects of VEGF signalling *in vivo* during malaria.

**Conclusions:**

Inhibition of VEGFR-2 signalling reduces intra-erythrocytic growth of *P. falciparum,* likely due to tyrosine kinase inhibition. Internalisation of VEGF in *P. falciparum-*infected red blood cells does not rely on VEGF receptors. The function of *in vivo* uptake of VEGF can be studied in rodent malaria models.

## Background

*Plasmodium falciparum* malaria is responsible for over one million deaths annually, caused by complications like severe anaemia and cerebral malaria (CM). The clinical outcome of malaria is influenced by host genetics and parasite characteristics [1-3].

Sequestration of parasitized red blood cells (PRBCs) in cerebral blood vessels, resulting in local hypoxia and neuronal damage, is a key event in the pathogenesis of CM [2]. The angiogenic and neuroprotective glycoprotein vascular endothelial growth factor (VEGF) can potentially be induced by these mechanisms. Indeed, it has been shown to be associated to malaria. In nonimmune travellers and Kenyan children with malaria, VEGF is increased in both brain tissue and blood [4,5]. Its release has mainly been linked to hypoxia [6] since its expression is stimulated via stabilization of hypoxia inducible factor (HIF)-1α [7]. Also inflammation results in increased VEGF expression [8], and it may be a non-specific response to severe disease [9]. In human CM, histopathological analyses *post mortem* as well as studies on cerebral blood flow in comatose patients strongly support localized cerebral hypoxia, hypoperfusion, or both [9,10]. HIF-1α, which has a short half-life, was undetectable in human brain tissue *post mortem*, but a HIF-1α associated protein, DEC-1 was upregulated in neurons [9]. Thus, sequestration of PRBCs followed by local cerebral hypoxia and accumulation of HIF-1α is a possible cause of VEGF production during CM.

There is an unexplained uptake of VEGF in a large fraction of PRBCs during both uncomplicated and severe malaria [9]. In fatal CM cases, the proportion of VEGF-positive PRBCs is proportional to the degree of parasite sequestration in cerebral blood vessels [9]. Addition of VEGF to *in vitro* cultured *P. falciparum* increases parasitaemia, implying that VEGF may be a trophic factor for the parasites [11]. VEGF uptake has been proposed to depend on VEGF-receptor-2 (VEGFR-2), since this receptor has been demonstrated on the red blood cell surface in serum-enriched cultures of *P. falciparum*[11]. Cultivation in serum-free medium reduced the trophic effect of VEGF concomitantly with a reduction of VEGFR-2 on red blood cells [11]. VEGFR-2, a tyrosine kinase, is the major mediator of the antiapoptotic, mitogenic, angiogenic, and permeability-enhancing effects of VEGF on endothelial cells in adult persons and the expression of VEGFR-2 is increased during hypoxia [7].

This study was performed to investigate, whether direct inhibition of VEGF by the monoclonal anti-VEGF antibody bevacizumab or inhibition of VEGF receptors would reduce *P. falciparum* growth and prevent uptake of VEGF into PRBCs. Furthermore the *in vivo* uptake of VEGF was tested in the rodent malaria strain *Plasmodium berghei* ANKA, which serves as a mouse model of CM.

## Methods

### *In vitro* culture of *Plasmodium falciparum*

*Plasmodium falciparum* strain 3D7 was cultured in human serum-enriched medium according to standard methods [12]. Briefly, the parasites were grown in culture flasks at 37°C at 4% haematocrit in HEPES-buffered RPMI 1640 medium (Gibco, Life Technologies, Paisley, UK) supplemented with 10% human serum (blood group O), 0.05 mg/ml gentamycin (Gibco), 0.18 mg/ml L-glutamine (Sigma-Aldrich) in an atmosphere of 5% O_2_, 5% CO_2_, and 90% N_2_. Throughout the study, parasites were subcultured by adding fresh group O red blood cells whenever parasitaemia reached 5%. Human blood was drawn from healthy volunteers after obtaining verbal informed consent. Under Danish regulations, this did not require approval from an ethics committee. To produce serum, blood was allowed to clot. After centrifugation serum was aspirated, immediately frozen, and stored at -20°C until used. All experiments were performed in triplicate and repeated at least three times, unless stated otherwise.

### Inhibition of VEGF, VEGFR-1 and VEGFR-2

At day 0, 50 μL of a healthy malaria culture with a haematocrit of 50% and a parasitaemia of 0.4% was added to 150 μL of culture medium in microtitre plates. Prior to seeding, PRBCs were enriched for ring stages by centrifugation on 5% sorbitol (Sigma-Adrich) as previously described [13]. Culture medium was carefully sampled and replaced by pre-warmed medium.

For direct VEGF inhibition, the humanized monoclonal anti-VEGF antibody bevacizumab (Avastin, Roche, Denmark) was added daily to the growth medium, resulting in the following concentrations in four different groups: 10 nM, 100 nM, 1,000 nM, and 10 μM. To allow for binding between bevacizumab and any VEGF in the growth medium, bevacizumab and growth medium were mixed at least one hour prior to addition to the culture. Growth medium with phosphate buffered saline (PBS) added instead of bevacizumab was used as control.

For VEGFR-1 inhibition, the anti-VEGFR-1 peptide which blocks the VEGF binding site on VEGFR-1 [14] (Anaspec, CA, USA) was added daily to the growth medium resulting in concentrations of 7 μM, 22 μM, 67 μM, and 200 μM respectively, at least one hour prior to addition to the culture. Growth medium with PBS added instead of anti-VEGFR-1 was used as control.

For VEGFR-2 inhibition, the VEGFR-2 inhibitor SU5416 (Tocris Bioscience, UK) was added daily to the growth medium, resulting in concentrations of 4 μM, 20 μM, 100 μM, and 500 μM respectively, at least one hour prior to addition to the culture. Growth medium with dimethyl sulphoxide (DMSO, Sigma-Aldrich, Denmark) added instead of SU5416 was used as control.

### Inhibition of tyrosine kinase activity

For inhibition of tyrosine kinase activity a broad-acting inhibitor, genistein (Sigma-Aldrich, Denmark) was used. Genistein was used at four concentrations: 1, 4, 16, and 64 μg/ml, which were prepared at least one hour prior to addition to the culture. Growth medium with DMSO added instead of genistein was used as control. These concentrations were also applied for growth inhibition assays.

### Immunocytochemistry

Thin blood smears of cultured blood were fixed in a mixture of 25% acetone (Merck Chemicals, Germany) and 75% absolute ethanol (Bie & Berntsen A/S, Denmark). After fixation, slides were permeabilized with 0.1% triton x-100 (Sigma-Aldrich, Denmark) and blocked with 5% bovine serum albumin (Sigma-Aldrich, Denmark) for one hour. Incubation for one hour with primary antibody (rabbit anti-VEGF Ab-1, ThermoScientific, Denmark) at a concentration of 0.01 mg/mL, and secondary antibody (goat anti-rabbit Alexa Fluor 488, Life Technologies, Denmark) at a concentration of 0.5 μg/mL, were followed by a nuclear stain (DAPI, Life Technologies). Samples without primary antibody served as negative controls. The negative control was further verified by pre-incubation where an excess (1.5x) of mouse VEGF (R&D Systems, UK) was added to the primary antibody 0.5 hours prior to incubating on the thin blood smears. Slides were covered with anti fade reagent (Slow Fade Gold anti fade reagent, Life Technologies) and cover slipped.

### Fluorescence microscopy

Light immunofluorescence microscopy photos were taken with an Olympus BX40 microscope equipped with an Olympus DP71 digital camera. DAPI (Olympus filter cube WU, excitation 330–385, emission 420) and fluorescein (3540B, excitation 482/35, emission 536/40, Olympus) filters were used. VIS (version 2.14.10.0) was used to acquire and save images. ImageJ (version 1.44i [15]) was used for further processing. Parasite development stage was determined by the number of parasite nuclei per red blood cell. Red blood cells with three or more distinguishable parasite nuclei were considered late stage.

### Confocal microscopy

Confocal immunofluorescence microscopy photos were taken with a Nikon TE 2000E Eclipse, with 360 numerical aperture, 1.4 Apoplan oil immersion objective lens, with gain adjusted for each laser (515/30, 605/75). Z-stacks were generated with Nikon EZ-C1 software with z-steps of 0.15 μm.

### Flow cytometry

Parasitaemia was determined daily by staining of samples with acridine orange (Sigma-Aldrich, Denmark) followed by flow cytometry as previously described [16]. Samples were processed on a FACSCanto flow cytometer (BD Biosciences, USA). Ten thousand events were recorded for each sample and the resulting scatter plots were analysed using the Weasel software (The Walter and Eliza Hall Institute of Medical Research, version 3.0).

For the detection of intracellular VEGF in PRBCs by flow cytometry at day 3, when late-stage parasites were predominant, cells were fixed in 1% paraformaldehyde (Ampliqon, Denmark) for 10 min at room temperature. After washing in PBS, the cells were permeabilized in 0.1% saponin (Sigma-Aldrich, Denmark) for 10 min. Intracellular VEGF was detected by incubating 5×10^5^ cells for 40 min with mouse anti-human VEGF conjugated to allophycocyanin (2 μg/ml, clone 23410, R&D Systems) according to manufacturer’s instructions. The reactivity was compared with a relevant isotype control (2 μg/ml, R&D Systems). Fifty thousand events were recorded using the 633 HeNe laser. The DNA binding dye Syto-9 (5 μM, Life Technologies) was added to the staining solution. This made it possible to discriminate PRBCs, which contain DNA, from nonparasitized red blood cells, which do not contain DNA. The precision of this detection of PRBCs was comparable with acridine orange (p > 0.61). The 488 nm Argon laser was used for Syto-9. Data analyses were carried out using FlowJo version 7.6.5 (Three Star Inc, OR, USA).

### Western blotting

At day 1, late-stage PRBCs were lysed in PhosphoSafe (Merck Chemicals, Germany) to preserve the current phosphorylation of proteins according to the manufacturer’s instructions. The lysates were stored at -20°C until western blotting was performed. Protein content was determined using the Lowry assay (DC protein assay, Bio-Rad, CA, USA). The proteins were separated on 10% polyacrylamide gels (BisTris mini, Life Technologies) at 150 V using MES buffer (Life Technologies). Twenty μg protein was loaded into each well and electrophoresis was carried out under denaturing and reducing conditions. Proteins were transferred to polyvinylidene membranes (ImmunBlot, Bio-Rad) at 30 V for one hour. The membranes were blocked with 5% bovine serum albumin (Sigma-Aldrich, Denmark) for one hour at room temperature before being incubated with an anti-phosphorylated tyrosine (pTyr) antibody at 4°C overnight (2000 × dilution, Cell Signaling Technology, MA, USA). The primary antibody was detected using a goat anti-mouse horseradish peroxidase-conjugated secondary antibody (Dako, Glostrup, Denmark). Bands were detected using SuperSignal West Femto Substrate (Pierce, Thermo Fischer, IL, USA). The bands were quantified using open-access Fiji software [17]. The bands were normalized to total protein content of the well by shortly staining the membrane in 0.25% Coomassie Blue and destaining overnight according to standard methods before scanning the membrane in epi-illumination mode (Chemidoc XRS, Bio-Rad). This experiment was performed three times in duplicate.

### Animal experiments

Female, pathogen-free, six- to eight-week old C57/Bl6 mice (Taconic, Ejby, Denmark) were infected by intraperitoneal (ip) injection of *P. berghei* ANKA (inoculum size = 10^4^ PRBCs). Animals were treated according to standard protocols and given food and water *ad libitum*. The experiment was approved by the Danish Animals Inspectorate (license number 2006/561-1128). On day 8, peripheral blood was collected for thin blood smears and processed for immunocytochemistry as described.

### Statistics

Data did not meet the criteria for using parametric tests. Kruskal-Wallis test was applied for comparing multiple groups and Mann–Whitney U test was used *post hoc*. A p-value less than 0.05 was considered statistically significant. All statistical analyses were performed with the R-commander software [18].

## Results

### VEGF uptake in PRBCs

**Figure 1:**
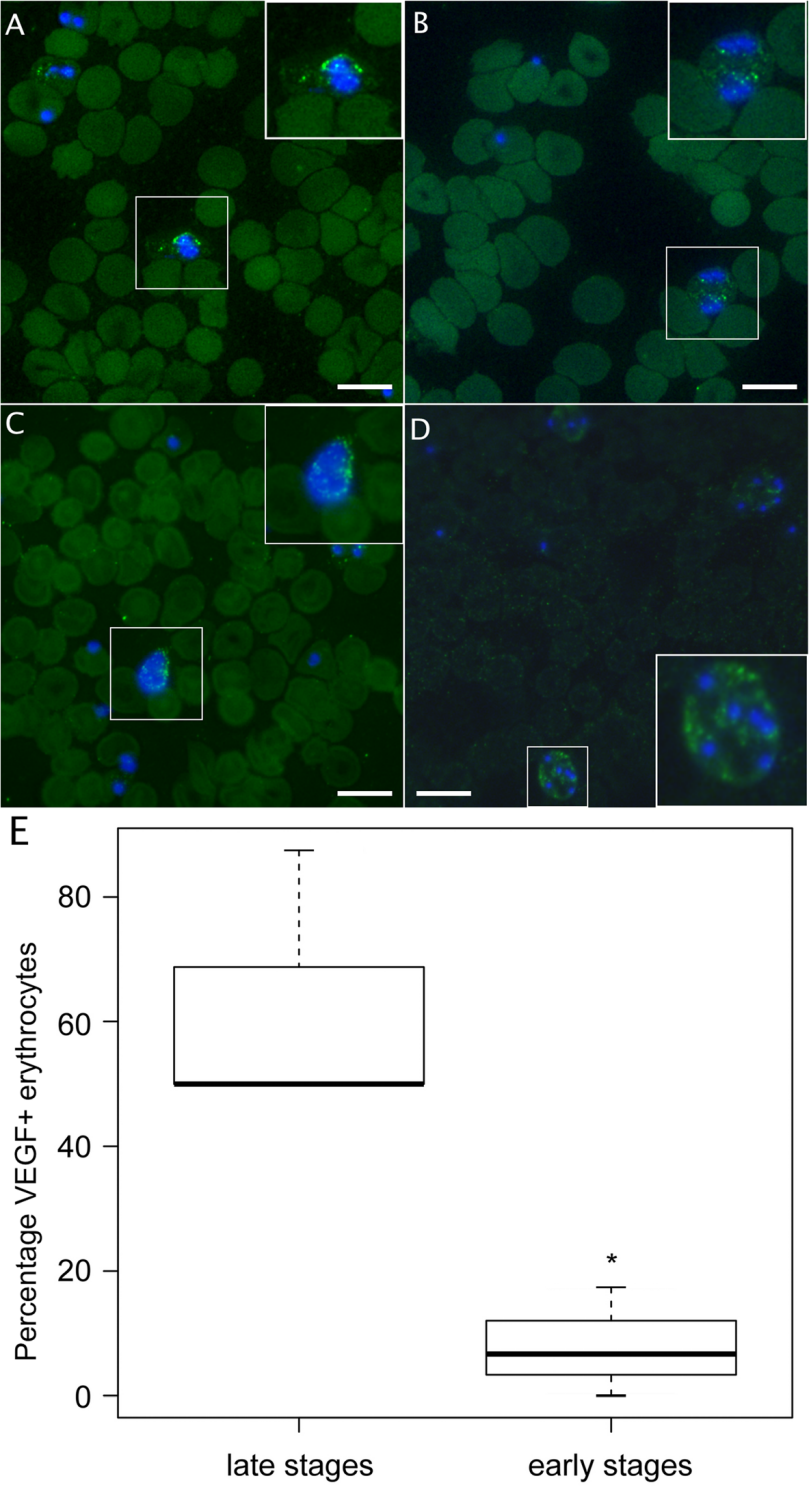
**Immunocytochemistry (ICC) of cultured human red blood cells infected with *Plasmodium falciparum *and peripheral blood smears from mice infected with *Plasmodium berghei *ANKA show uptake of VEGF into late stage parasites, not inhibited by blocking VEGFR-2 or VEGF.** Fluorescence microscopy of thin blood smears after culture of human blood infected with *P. falciparum* with no inhibition **(A)**, SU5416 **(B)**, or bevacizumab **(C)**. DNA is stained blue by DAPI, VEGF is stained green. Uninfected red blood cells and most early stage parasitized red blood cells (containing a maximum of two DNA fragments) did not stain positive for VEGF. Inlets present 1.5 x enlargement of marked area on the slide. Bar size = 10 μm. Thin blood smears taken from mice infected with *P. berghei* ANKA **(D)** showed similar staining compared to culture of human blood. Inlet presents 2 x enlargement of marked area on the slide. Bar size = 10 μm. Manual counting of parasitized red blood cells showed a significant increase in VEGF uptake in late stage PRBCs (containing more than two DNA fragments) compared to early stages (p = 0.038, **E**).

Culture of *P. falciparum* in serum-enriched growth medium resulted in uptake of VEGF into PRBCs, determined by immunocytochemistry on day 6 after the first addition of serum (Figure 1). Neither uninfected red blood cells nor free parasites stained positive for VEGF (Figure 1). This was confirmed by flow cytometry. Confocal microscopy at high magnification showed that VEGF was located intracellularly, in relation to the parasites (Additional file 1: Movie 1). Pre-incubation of the primary anti-VEGF antibody with an excess of murine VEGF prior to incubation almost completely abolished staining for VEGF. The incidence of VEGF uptake was significantly increased in late stages of the malaria parasite (with detection of VEGF in 60.7% of PRBCs), compared to early stages (VEGF in 6.7% of PRBCs, p = 0.038, Figure 1E).

### Direct inhibition of VEGF by bevacizumab does not reduce parasitaemia

**Figure 2:**
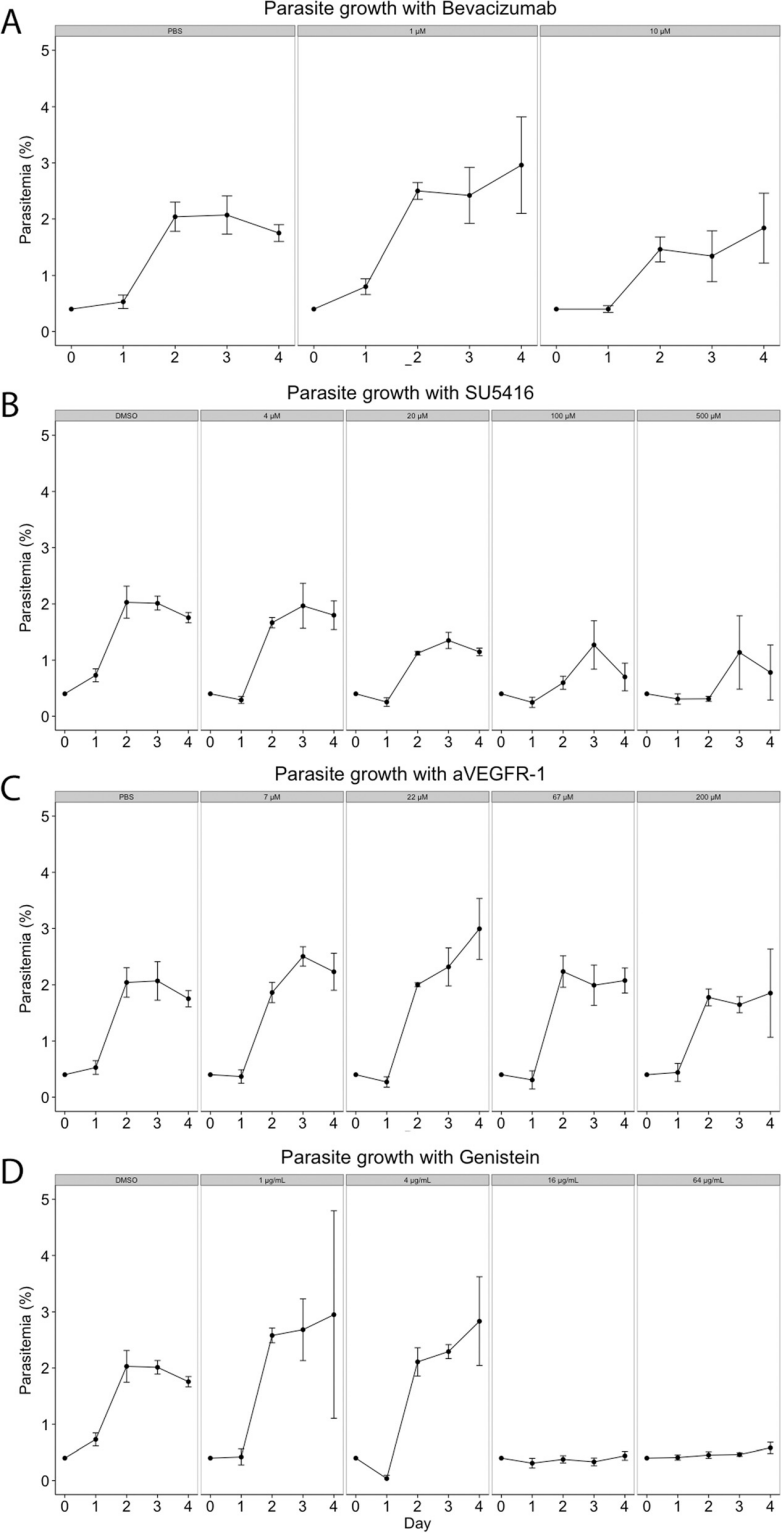
**Inhibition of VEGFR-2 but not VEGFR-1 or VEGF reduces parasitaemia in cultures of *Plasmodium falciparum*.** Unspecific inhibition of tyrosine kinases also reduces parasitaemia in cultures. Growth curves of cultures of human blood cells infected with *P. falciparum*, in the presence of bevacizumab **(A)**, SU5416 **(B)**, anti-VEGFR-1 **(C)**, or genistein **(D)** in increasing concentrations. Controls contain only PBS or DMSO respectively. Inhibition of VEGF by bevacizumab **(A)** did not affect parasitaemia, whereas inhibition of VEGFR-2 by SU5416 **(B)** resulted in a significantly reduced parasitaemia in a dose-dependent manner compared to DMSO controls. Inhibition of VEGFR-1 by anti-VEGFR-1 **(C)** did not replicate this effect and had no effect on parasitaemia. Addition of the unspecific tyrosine kinase inhibitor genistein **(D)** resulted in a significant reduction in parasitaemia compared to DMSO.

Direct inhibition of VEGF in the growth medium was obtained by adding the humanized monoclonal anti-VEGF antibody bevacizumab. Flow cytometric analyses showed that parasitaemia did not change significantly compared to PBS-treated controls on any day until the experiment was ended (p > 0.06, Figure 2A).

### Inhibition of VEGFR-2 by SU5416 significantly reduces parasitaemia

Inhibition of VEGFR-2 was obtained by addition of the VEGFR-2 inhibitor SU5416 to the growth medium. This treatment resulted in a significant and dose dependent decrease in parasitaemia as compared to DMSO treated controls from day 2 and onwards (p < 0.05, Figure 2B). At day 2 all doses but 4 μM resulted in significant growth retardation (p < 0.01). On day 3 and 4 only the two highest doses (100 and 500 μM) significantly reduced growth (p < 0.01), while at 20 μM it was statistically indistinguishable from DMSO (p > 0.12). Four μM SU5416 did not reduce the parasitaemia in culture compared to control during experiments (p > 0.07, Figure 2B).

### Direct inhibition of VEGFR-1 does not reduce parasitaemia

Direct inhibition of VEGFR-1 in the growth medium was obtained by adding a peptide binding to VEGFR-1 thereby inhibiting its interaction with VEGF. This treatment did not significantly affect parasite growth compared to PBS-treated controls on any day of the experiment (p > 0.06, Figure 2C).

### Treatment with bevacizumab, SU5416 or anti-VEGFR-1 does not cause reduced VEGF accumulation

**Figure 3:**
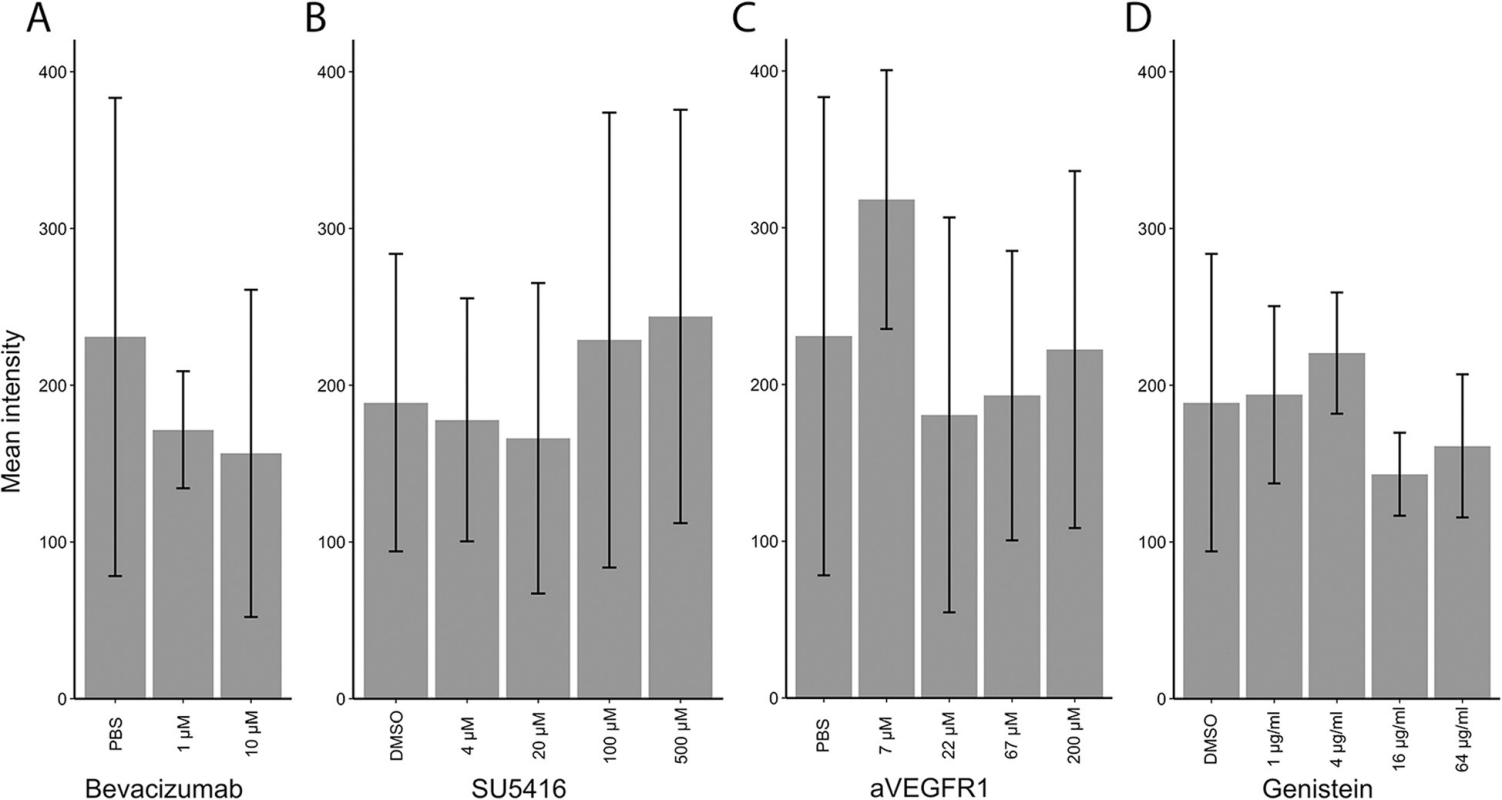
**Intracellular VEGF content in parasitized red blood cells (PRBCs) is not affected by inhibiting VEGF, VEGFR-2, VEGFR-1, or tyrosine kinases.** Median fluorescence intensity of PRBCs collected at day 3 from cultures of *P. falciparum* after staining for intracellular VEGF, determined by flow cytometry. Neither inhibition of VEGF by bevacizumab **(A)**, nor specific inhibition of VEGFR-2 by SU5416 **(B)**, or VEGFR-1 by anti-VEGFR-1 peptide **(C)** significantly altered the accumulation of VEGF in PRBCs. Broad inhibition of tyrosine kinases by adding genistein **(D)** had no effect on accumulation of VEGF in PRBCs.

The intracellular accumulation of VEGF was studied in parallel with the progression of parasitaemia by flow cytometry analyses of the median fluorescence intensity after staining. This did not reveal any reduction in VEGF accumulation after four days of treatment with bevacizumab (p > 0.51), SU5416 (p > 0.06), or anti-VEGFR-1 (p > 0.34) compared to DMSO and PBS controls respectively (Figure 3A-C). The flow cytometric results were confirmed by immunocytochemistry, showing VEGF staining of PRBCs despite treatment with SU5416 (Figure 1B) or bevacizumab (Figure 1C).

### DNA content of PRBCs upon SU5416 and bevacizumab stimulation

Previous studies have indicated that VEGF is trophic for parasite growth [11]. One mechanism by which SU5416 could reduce parasite growth could be through reduction of the number of merozoites per schizont, which has previously been shown to affect parasite growth rate [19], thus reducing the number of newly infected red blood cells after schizont rupture. However, analyses of flow cytometric data (median fluorescence intensity of DNA binding dye Syto-9) did not support that either SU5416 (p = 0.054) or bevacizumab (p = 0.095) reduced the DNA content of the PRBCs.

### Genistein dose-dependently decreases parasitaemia

Since no association between inhibition of parasite growth and intracellular VEGF was found, an alternative anti-parasitic mechanism of SU5416 was sought. SU5416 inhibits VEGFR-2 signalling as a tyrosine kinase inhibitor. Therefore, the effect of genistein, a broad-acting tyrosine kinase inhibitor, on intra-erythrocytic growth of *P. falciparum* was tested. As seen in Figure 2D genistein inhibited growth significantly in a dose-dependent manner (p < 0.02), while not preventing VEGF accumulation in PRBCs (Figure 3D).

### SU5416 and genistein dose-dependently reduce phosphorylation of tyrosine

**Figure 4:**
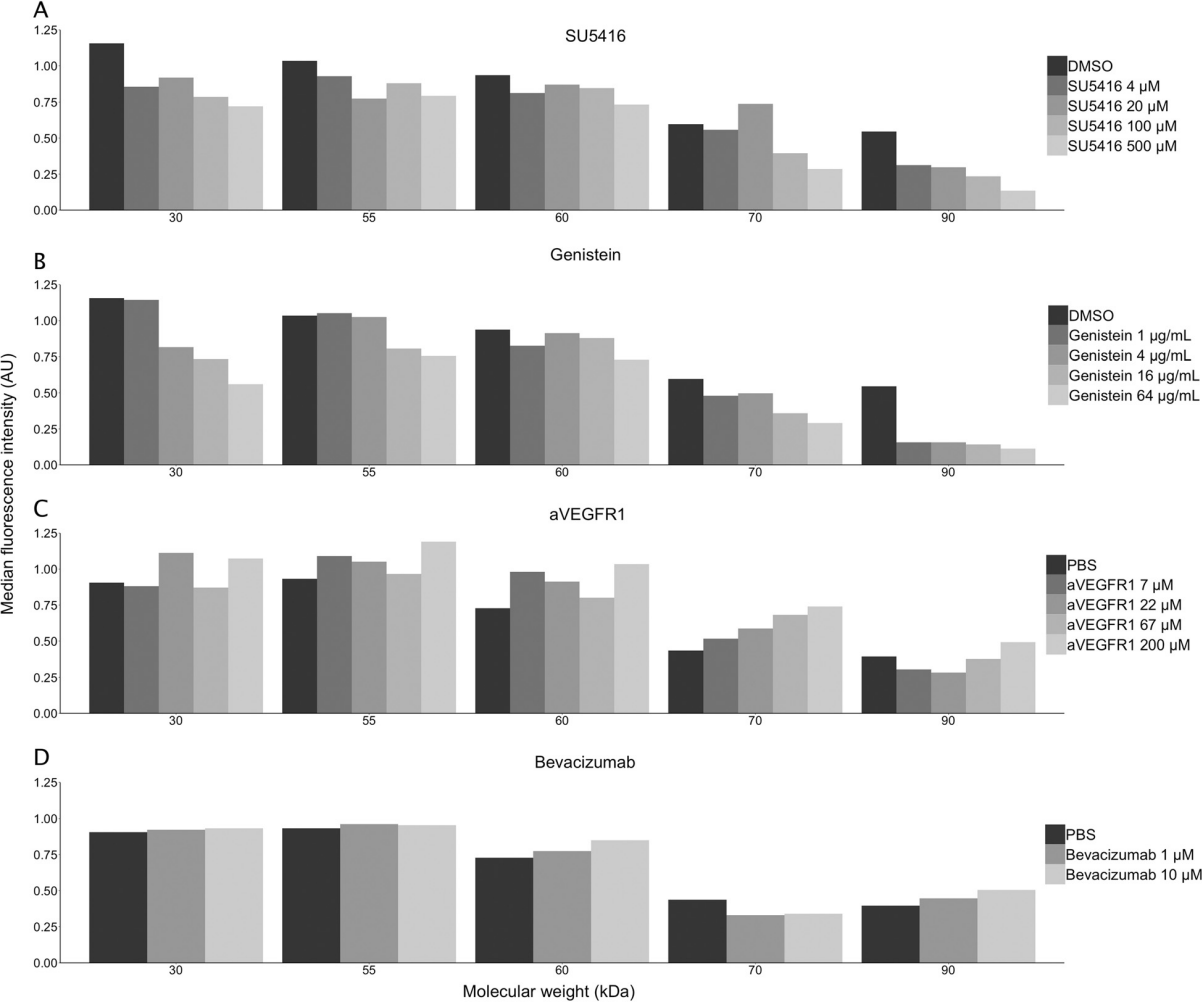
**Specific or unspecific inhibition of VEGFR-2, by addition of SU5416 or genistein to cultures of *Plasmodium falciparum*, reduces phosphorylation of several tyrosine kinases in red blood cells.** This result is not replicated by addition of anti-VEGFR-1 or bevacizumab. Median intensity of bands, obtained by staining western blots of red blood cells after culture, for several phosphorylated tyrosine kinases, with molecular weights of 30, 55, 60, 70, and 90 kDa respectively. Cultures were performed in duplicate with the addition of increasing concentrations of SU5416, genistein, anti-VEGFR-1, or bevacizumab. SU5416 **(A)** and genistein **(B)** inhibited several of the tested tyrosine kinases in a dose dependent manner. This was not the case for anti-VEGFR-1 **(C)** or bevacizumab **(D)**.

The effect of SU5416 on tyrosine phosphorylation was investigated and compared with the other treatments applied. SU5416 and genistein dose-dependently reduced tyrosine phosphorylation in PRBCs (Figure 4A-B), whereas anti-VEGFR-1 peptide and bevacizumab did not show any effect on this parameter (Figure 4C-D).

### Uptake of VEGF into red blood cells infected with *Plasmodium berghei* ANKA

The murine malaria parasite *P. berghei* ANKA, when infecting mice *in vivo*, internalized VEGF similarly to the *in vitro* experiments. Peripheral blood smears, collected after eight days of infection, showed a marked uptake of VEGF into PRBCs, primarily in schizonts (Figure 1D). Confocal microscopy showed that VEGF was located intracellularly (Additional file 2: Movie 2). Analogous to the *in vitro* results with *P. falciparum*, non-PRBCs and free parasites did not stain for VEGF (Figure 1D).

## Discussion

The presented results strengthen the evidence of VEGF uptake during *in vitro* culture of *P. falciparum*[11] and suggest that uptake of VEGF depends on parasite maturity. However, the mechanism behind this uptake remains unclear. This may not be an active process since applying an excess level of anti-VEGF antibody neither affects growth nor intracellular VEGF levels. A dedicated VEGF transporter would likely be hindered by VEGF being of different size or having potential binding sites blocked by the antibody. It is known that the red blood cell membrane in late-stage parasites becomes leaky [20], which could augment internalization of VEGF. Similarly, there is an intracellular accumulation of VEGFR-1 in late-stage parasites (unpublished observation). Previous reports suggest that VEGF acts as a trophic factor for parasite growth since addition of VEGF to an *in vitro* parasite culture increased parasitaemia and partly rescued the culture from pre-treatment with artesunate [11]. As it was not possible to reduce intracellular VEGF, this effect could not be assessed further.

Besides being a trophic factor, a possible advantage of VEGF accumulation could be the removal of VEGF from the micro-environment in small brain vessels. Neuronal and epithelial VEGF production is increased during cerebral hypoxia, resulting in nitric oxide (NO)-dependent vasodilation, increased angiogenesis, and thus increased oxygen delivery to the tissue [6]. Because of the micro-aerophilic nature of *P. falciparum*[21], these changes are potentially detrimental to the parasite, making it a good survival strategy to remove VEGF.

Avoidance of the host immune system is a third possible rationale for VEGF uptake. VEGF is known to stimulate monocyte and T-cell chemotaxis [22,23]. Circulating monocytes and resident macrophages contribute to parasite clearance by phagocytizing PRBCs, and are present in increased numbers in cerebral vessels with sequestration [24]. Thus, removal of VEGF from the cerebral micro-environment could reduce local host immune responses and allow parasite sequestration in the cerebral microvasculature.

Confocal microscopy proved that VEGF was located intracellularly in the PRBCs. This excludes that positive staining for VEGF in PRBCs was due to adsorption onto the PRBC surface rather than absorption. Confocal microscopy did not allow to determine the precise intracellular distribution of VEGF. However it did show that at least some VEGF is present in the parasitophorous vacuoles.

Both microscopy and flow cytometry showed that VEGF uptake was not significantly reduced despite VEGF immobilization by a monoclonal antibody, and neither by VEGFR-1 nor VEGFR-2 inhibition. However, inhibition of VEGFR-2 signalling significantly decreased parasitaemia in this study. The antiparasitic action of VEGFR-2 inhibition despite unchanged VEGF accumulation suggests that this effect is not directly related to uptake of VEGF. Instead, SU5416 possibly inhibits the intracellular downstream signalling cascade relating to VEGFR-2 activation. VEGFR-2 is a receptor tyrosine kinase. It exerts its effects, e.g. angiogenesis and tumour growth, via intracellular phosphorylation of tyrosine [25,26]. Indeed, *P. falciparum* also relies on tyrosine kinase activity [27]. Co-culturing *P. falciparum* with genistein, a broad-acting tyrosine kinase inhibitor, dose-dependently reduced parasite growth, as previously shown [28,29]. Western blotting of phosphotyrosine levels in late-stage parasites supported the concept that SU5416 directly inhibited parasite tyrosine kinases, as several loci had reduced phosphotyrosine levels. Comparable results were obtained with genistein though it seemed to inhibit a broader range of kinases. Additional research is needed to further assess the association between tyrosine kinase inhibition and the anti-parasitic effect of SU5416 and genistein.

Many studies on the pathogenesis of CM are performed in a mouse model, infected with the rodent *Plasmodium* strain *P. berghei* ANKA, because of its accessibility as well as clinical and histopathological similarities to human CM [30]. The presented results show *in vivo* uptake of VEGF into murine PRBCs infected with *P. berghei* ANKA, similar to human PRBCs. VEGF was located intracellularly in late-stage PRBCs. Thus future studies of VEGF inhibitors may be performed *in vivo* using this experimental model.

In summary, inhibition of VEGFR-2 signalling reduces intra-erythrocytic growth of *P. falciparum*, but this reduction does not correlate with intracellular levels of VEGF. Rather, it may depend on inhibition of parasite tyrosine kinase activity. The effect of VEGF on parasite growth remains to be clarified. Since VEGF also accumulates in late-stage *P. berghei* ANKA PRBCs *in vivo*, the relevance of VEGF signalling may be addressed in experimental infections.

## Competing interests

The authors declare that they have no competing interests.

## Authors’ contributions

CH and NH designed and carried out the experiments, performed the statistical analysis and drafted the manuscript. TS and JK participated in the design, coordination, and interpretation of the study. All authors read and approved the final manuscript.

## Supplementary Material

Additional file 1: Movie 1 Z-stack obtained by confocal microscopy showing intracellular VEGF in cultured human red blood cells infected with *Plasmodium falciparum.* DNA is stained blue and VEGF is stained green by immunocytochemistry. The stack shows VEGF located intracellularly. The z-stack was generated with Nikon EZ-C1 software with z-steps of 0.15 μm. Click here for file

Additional file 2: Movie 2 Z-stack obtained by confocal microscopy showing intracellular VEGF in murine red blood cells infected with *Plasmodium berghei* ANKA during experimental CM. DNA is stained blue and VEGF is stained green by immunocytochemistry. The stack confirms that VEGF is located intracellularly and shows a similar pattern of distribution as cultured human red blood cells infected with *P. falciparum*. The z-stack was generated with Nikon EZ-C1 software with z-steps of 0.15 μm. Click here for file

## References

[B1] Wassmer SC Moxon CA Taylor T Grau GE Molyneux ME Craig AG Vascular endothelial cells cultured from patients with cerebral or uncomplicated malaria exhibit differential reactivity to TNF Cell Microbiol 2011 13 198 209 10.1111/j.1462-5822.2010.01528.x 21029292 PMC3041929

[B2] Turner L Lavstsen T Berger SS Wang CW Petersen JE Avril M Brazier AJ Freeth J Jespersen JS Nielsen MA Magistrado P Lusingu J Smith JD Higgins MK Theander TG Severe malaria is associated with parasite binding to endothelial protein C receptor Nature 2013 498 502 505 10.1038/nature12216 23739325 PMC3870021

[B3] Murphy SC Breman JG Gaps in the childhood malaria burden in Africa: cerebral malaria, neurological sequelae, anemia, respiratory distress, hypoglycemia, and complications of pregnancy Am J Trop Med Hyg 2001 64 57 67 11425178 10.4269/ajtmh.2001.64.57

[B4] Deininger MH Winkler S Kremsner PG Meyermann R Schluesener HJ Angiogenic proteins in brains of patients who died with cerebral malaria J Neuroimmunol 2003 142 101 111 10.1016/S0165-5728(03)00250-9 14512169

[B5] Casals-Pascual C Idro R Gicheru N Gwer S Kitsao B Gitau E Mwakesi R Roberts DJ Newton CR High levels of erythropoietin are associated with protection against neurological sequelae in African children with cerebral malaria Proc Natl Acad Sci U S A 2008 105 2634 2639 10.1073/pnas.0709715105 18263734 PMC2268188

[B6] Ferrara N Davis-Smyth T The biology of vascular endothelial growth factor In Endocr Rev 1997 18 4 25 10.1210/edrv.18.1.0287 9034784

[B7] Hellwig-Burgel T Stiehl DP Wagner AE Metzen E Jelkmann W Review: hypoxia-inducible factor-1 (HIF-1): a novel transcription factor in immune reactions J Interferon Cytokine Res 2005 25 297 310 10.1089/jir.2005.25.297 15957953

[B8] Yano K Liaw PC Mullington JM Shih SC Okada H Bodyak N Kang PM Toltl L Belikoff B Buras J Simms BT Mizgerd JP Carmeliet P Karumanchi SA Aird WC Vascular endothelial growth factor is an important determinant of sepsis morbidity and mortality J Exp Med 2006 203 1447 1458 10.1084/jem.20060375 16702604 PMC2118329

[B9] Medana IM Day NP Roberts R Sachanonta N Turley H Pongponratn E Hien TT White NJ Turner GD Induction of the vascular endothelial growth factor pathway in the brain of adults with fatal falciparum malaria is a non-specific response to severe disease Histopathology 2010 57 282 294 10.1111/j.1365-2559.2010.03619.x 20716170 PMC2941727

[B10] Warrell DA White NJ Veall N Looareesuwan S Chanthavanich P Phillips RE Karbwang J Pongpaew P Krishna S Cerebral anaerobic glycolysis and reduced cerebral oxygen transport in human cerebral malaria Lancet 1988 2 534 538 2900921 10.1016/s0140-6736(88)92658-x

[B11] Sachanonta N Medana IM Roberts R Jones M Day NP White NJ Ferguson DJ Turner GD Pongponratn E Host vascular endothelial growth factor is trophic for Plasmodium falciparum-infected red blood cells Asian Pac J Allergy Immunol 2008 26 37 45 18595528

[B12] Trager W Jenson JB Cultivation of malarial parasites Nature 1978 273 621 622 10.1038/273621a0 351412

[B13] Lambros C Vanderberg JP Synchronization of Plasmodium falciparum erythrocytic stages in culture J Parasitol 1979 65 418 420 10.2307/3280287 383936

[B14] Bae DG Kim TD Li G Yoon WH Chae CB Anti-flt1 peptide, a vascular endothelial growth factor receptor 1-specific hexapeptide, inhibits tumor growth and metastasis Clin Cancer Res 2005 11 2651 2661 10.1158/1078-0432.CCR-04-1564 15814646

[B15] Schneider CA Rasband WS Eliceiri KW NIH Image to ImageJ: 25 years of image analysis Nat Methods 2012 9 671 675 10.1038/nmeth.2089 22930834 PMC5554542

[B16] Hein-Kristensen L Wiese L Kurtzhals JA Staalsoe T In-depth validation of acridine orange staining for flow cytometric parasite and reticulocyte enumeration in an experimental model using *Plasmodium berghei* Exp Parasitol 2009 123 152 157 10.1016/j.exppara.2009.06.010 19545567

[B17] Schindelin J Arganda-Carreras I Frise E Kaynig V Longair M Pietzsch T Preibisch S Rueden C Saalfeld S Schmid B Tinevez JY White DJ Hartenstein V Eliceiri K Tomancak P Cardona A Fiji: an open-source platform for biological-image analysis Nat Methods 2012 9 676 682 10.1038/nmeth.2019 22743772 PMC3855844

[B18] R Core Team R: A language and environment for statistical computing 2014 Vienna, Austria http://www.R-project.org

[B19] Reilly HB Wang H Steuter JA Marx AM Ferdig MT Quantitative dissection of clone-specific growth rates in cultured malaria parasites Int J Parasitol 2007 37 1599 1607 10.1016/j.ijpara.2007.05.003 17585919 PMC2268714

[B20] Kutner S Baruch D Ginsburg H Cabantchik ZI Alterations in membrane permeability of malaria-infected human erythrocytes are related to the growth stage of the parasite Biochim Biophys Acta 1982 687 113 117 10.1016/0005-2736(82)90178-X 7041976

[B21] Scheibel LW Ashton SH Trager W *Plasmodium falciparum*: microaerophilic requirements in human red blood cells Exp Parasitol 1979 47 410 418 10.1016/0014-4894(79)90094-8 36286

[B22] Clauss M Gerlach M Gerlach H Brett J Wang F Familletti PC Pan YC Olander JV Connolly DT Stern D Vascular permeability factor: a tumor-derived polypeptide that induces endothelial cell and monocyte procoagulant activity, and promotes monocyte migration J Exp Med 1990 172 1535 1545 10.1084/jem.172.6.1535 2258694 PMC2188755

[B23] Barleon B Hauser S Schollmann C Weindel K Marme D Yayon A Weich HA Differential expression of the two VEGF receptors flt and KDR in placenta and vascular endothelial cells J Cell Biochem 1994 54 56 66 10.1002/jcb.240540107 8126087

[B24] Pongponratn E Turner GD Day NP Phu NH Simpson JA Stepniewska K Mai NT Viriyavejakul P Looareesuwan S Hien TT Ferguson DJ White NJ An ultrastructural study of the brain in fatal *Plasmodium falciparum* malaria Am J Trop Med Hyg 2003 69 345 359 14640492

[B25] Fong TA Shawver LK Sun L Tang C App H Powell TJ Kim YH Schreck R Wang X Risau W Ullrich A Hirth KP McMahon G SU5416 is a potent and selective inhibitor of the vascular endothelial growth factor receptor (Flk-1/KDR) that inhibits tyrosine kinase catalysis, tumor vascularization, and growth of multiple tumor types Cancer Res 1999 59 99 106 9892193

[B26] Mendel DB Laird AD Smolich BD Blake RA Liang C Hannah AL Shaheen RM Ellis LM Weitman S Shawver LK Cherrington JM Development of SU5416, a selective small molecule inhibitor of VEGF receptor tyrosine kinase activity, as an anti-angiogenesis agent Anticancer Drug Res 2000 15 29 41 10888034

[B27] Solyakov L Halbert J Alam MM Semblat JP Dorin-Semblat D Reininger L Bottrill AR Mistry S Abdi A Fennell C Holland Z Demarta C Bouza Y Sicard A Nivez MP Eschenlauer S Lama T Thomas DC Sharma P Agarwal S Kern S Pradel G Graciotti M Tobin AB Doerig C Global kinomic and phospho-proteomic analyses of the human malaria parasite *Plasmodium falciparum* Nat Commun 2011 2 565 22127061 10.1038/ncomms1558

[B28] Dluzewski AR Garcia CR Inhibition of invasion and intraerythrocytic development of Plasmodium falciparum by kinase inhibitors Experientia 1996 52 621 623 10.1007/BF01969742 8698101

[B29] Kraft C Jenett-Siems K Siems K Gupta MP Bienzle U Eich E Antiplasmodial activity of isoflavones from *Andira inermis* J Ethnopharmacol 2000 73 131 135 10.1016/S0378-8741(00)00285-3 11025148

[B30] Hunt NH Grau GE Engwerda C Barnum SR der HH V Hansen DS Schofield L Golenser J Murine cerebral malaria: the whole story Trends Parasitol 2010 26 272 274 10.1016/j.pt.2010.03.006 20382078

